# Arthritis and Diagnostics in Lyme Disease

**DOI:** 10.3390/tropicalmed6010018

**Published:** 2021-01-29

**Authors:** Javier A. Quintero, Raluchukwu Attah, Reena Khianey, Eugenio Capitle, Steven E. Schutzer

**Affiliations:** 1 Division of Rheumatology, Department of Medicine, Rutgers New Jersey Medical School, Newark, NJ 07103, USA; 2 Department of Internal Medicine, Rutgers New Jersey Medical School, Newark, NJ 07103, USA; rca56@njms.rutgers.edu; 3 Division of Rheumatology, Allergy and Immunology, Department of Medicine, Rutgers New Jersey Medical School, Newark, NJ 07103, USA; khianere@njms.rutgers.edu (R.K.); capitlem@njms.rutgers.edu (E.C.); 4 Division of Allergy and Immunology, Department of Medicine, Rutgers New Jersey Medical School, Newark, NJ 07103, USA

**Keywords:** Lyme disease, Lyme arthritis, *Borrelia burgdorferi*, inflammatory arthritis, antibiotic refractory arthritis, disease modifying antirheumatic drugs, tumor necrosis factor inhibitors, antibodies, indirect testing, direct testing

## Abstract

The diagnosis of Lyme disease, caused by *Borrelia burgdorferi,* is clinical but frequently supported by laboratory tests. Lyme arthritis is now less frequently seen than at the time of its discovery. However, it still occurs, and it is important to recognize this, the differential diagnoses, and how laboratory tests can be useful and their limitations. The most frequently used diagnostic tests are antibody based. However, antibody testing still suffers from many drawbacks and is only an indirect measure of exposure. In contrast, evolving direct diagnostic methods can indicate active infection.

## 1. Introduction

Lyme disease (LD) is the most common vector-borne disease in the United States (US). It was initially defined in 1977 with the report of multiple cases of seemingly inflammatory arthritis in adults and children from three contiguous towns, including Lyme and Old Lyme Connecticut, US [1]. There are now more than 300,000 cases each year in the United States [2]. The Swiss-born scientist Dr. Wilhelm Burgdorfer is credited with isolating the pathogenic bacteria behind LD, *Borrelia burgdorferi*, in 1982 after receiving ticks from Dr. Jorge Benach [3]. 

The clinical manifestations of LD have been traditionally classified into three distinct stages; however, we now know that manifestations described for each stage may occur simultaneously or at different times. For the purpose of this paper, we will refer to them as stages: early localized stage, early disseminated stage and late disseminated stage. The skin is the most frequently observed system, but the nervous system, joints, and the heart can also be involved, and as such, a patient with LD may undergo evaluation by multiple specialists, such as neurologists, rheumatologists, orthopedists and cardiologists. Noting that Lyme arthritis is now less commonly seen, perhaps due to increased recognition of earlier stages of LD, it is meaningful to review certain clinical distinctions that are particular to this form of inflammatory arthritis; particularly from the point of view of specialists in musculoskeletal diseases such as rheumatologists. On the other hand, we find that all physicians in our community can also benefit by reflecting upon the strengths and limitations of current diagnostic tests in LD. It is important to note that we write this from a US-centric perspective where Lyme arthritis is more common, though it still shares some features with European forms of LD.

## 2. Lyme Arthritis

Lyme arthritis (LA), a feature of the late disseminated stage, is now less common than when LD was discovered [1,2,3,4]. LA may occur in approximately 60% of untreated patients, as was seen when LD was initially recognized in the 1970s. Among 107,272 reported cases in the US from 2008 to 2015, approximately 28% of patients reported symptoms of arthritis [2]. It manifests as a mono-arthritis of the knee or an asymmetric oligo-arthritis that may present with joint swelling, warmth and erythema [4,5,6]. LA is frequently described as swelling and articular stiffness that is out of proportion to the degree of joint pain [5,6]. Polyarthralgia may be seen in acute stages of LD, but polyarthritis during the disseminated stage can occur in 20% of untreated patients [4]. The knees are the most commonly affected joint in LA, seen in 80% of patients, but other large joints such as the shoulders, hips and elbows can be involved [4,5,6]. Eighteen percent of untreated patients may experience arthralgias without the development of actual frank arthritis [4]. Periarticular manifestations such as bursitis, tendinitis, and enthesitis can be seen, but may occur during any stage of LD or during inter-critical periods of recurrent flares of arthritis [4,5]. Axial and sacroiliac joint involvement is not a typical feature of LA [4,5]. Without treatment, arthritis may be recurrent, with episodes of synovitis lasting weeks to months followed by quiescent periods. However, these flares of arthritis often decrease in frequency and severity and may self-resolve over time, even without treatment [4,7]. Diagnosis of LA can be supported by the detection of antibodies against *B. burgdorferi* in the serum. Detection of *B. burgdorferi* DNA by polymerase chain reaction (PCR) in synovial fluid or synovium can also assist in diagnosis, but the rate of positive testing can range from 40% to 95% [8]. Remnant borrelial DNA may also persist in synovial fluid after adequate treatment, so synovial PCR is not an obligatory marker of active infection [8].

### 2.1. Lyme Arthritis and Differential Diagnoses

Other rheumatic conditions, such as reactive arthritis in adults or juvenile idiopathic arthritis in children, will share similar musculoskeletal manifestations with LA. Clinical history and serological evaluation for borrelial antibodies may help differentiate LA from these other chronic rheumatic disorders. Table 1 lists some common differential diagnoses. Usually, acute mechanical injury from trauma or sports injury is the first diagnostic consideration. Defining the clinical pattern of synovitis can also help distinguish LA from rheumatoid arthritis, which would present as a chronic symmetric polyarthritis involving small joints, frequently of the hands and wrists. Some features of LA may be suggestive of septic arthritis, especially in children, which often presents with a more clinically impressive arthritis than adults, and where joint pain and swelling may be accompanied by fever and elevated serum and synovial fluid white blood cell counts (WBC), as well as an elevation in inflammatory biomarkers such as erythrocyte sedimentation rate and c-reactive protein. Synovial fluid analysis in LA may vary but will show approximately 25,000 WBCs/mm^3^ (ranging from 500 to 100,000 WBCs/mm^3^) with neutrophilic predominance [5,6]. Myofascial pain syndromes such as fibromyalgia are also frequently misdiagnosed as LD, but clinical evidence of inflammatory arthritis is not a feature of fibromyalgia. It is easy to understand why other periarticular manifestations of LD can be confused with fibromyalgia, but a thorough clinical history describing the pattern of joint involvement supported by an adequate interpretation of laboratory testing is essential for differentiation between these two entities.

### 2.2. Treatment of Lyme Arthritis

Treatment options for adults with LA include oral doxycycline, amoxicillin, or cefuroxime for 28 days [9]. For children less than 8 years old, amoxicillin or cefuroxime had been preferred since doxycycline was thought to theoretically result in discoloration of teeth or gum hypoplasia in this cohort. However, since there is no clear evidence that a short course of treatment would have this effect in children, the American Academy of Pediatrics (AAP) have revised their stance on this matter and now allow up to 21 days of treatment with doxycycline [10,11]. Patients with mild persistent arthritis, despite completing an initial treatment course, can be re-treated with a second course of 28 days of oral antibiotics. Patients with moderate to severe recurrent arthritis can be treated with a 2 to 4-week course of IV ceftriaxone [8,9]. Less than 10% of patients may develop persistent synovitis despite multiple courses of antibiotics, a clinical entity known as “antibiotic-refractory arthritis”.

For patients with antibiotic-refractory arthritis and negative synovial PCR, no further antibiotics are recommended [8,9]. Treatment with non-steroidal anti-inflammatories (NSAIDs) or disease modifying antirheumatic drugs (DMARDs), such as hydroxychloroquine or methotrexate and even tumor necrosis factor (TNF) inhibitors, may be used in these patients [9,12]. Administration of intraarticular corticosteroids is another option which can be considered after at least two full courses of antibiotics and confirmation of a negative synovial PCR [8,12]. Ultimately, for any persistent synovitis despite treatment, or an inability to tolerate typical treatment options, arthroscopic synovectomy may be an alternative course of action [9,12].

## 3. Diagnostic Methods in Lyme Disease

Diagnostic consideration of LD begins with an adequate clinical suspicion and an appropriately high pre-test probability based on known epidemiological risk factors. The diagnosis of untreated and first exposure during the localized or disseminated stages may be supported by serological detection of antibodies directed against *B. burgdorferi*. However, a single antibody test is not a marker of active infection. In LD, there is a relatively long lag period of approximately 3 weeks before a serological test actually reaches a threshold of positivity, so early testing during this seronegative period, even in a true case, may lead to a high rate of false negatives. Other patients may prove to be seronegative because the presence of their anti-borrelial antibodies is found in the form of immune complexes with borrelial antigens, thus masking serological detection by conventional immunoassay [13]. In addition, early antibiotic therapy may abrogate the detection of antibodies by a conventional assay.

Diagnosis during the early localized stage depends on clinical evidence of the classic ring-within-a-ring, especially when expanding, erythema migrans (EM) skin lesion and possible exposure to a tick bite. Recognition of EM in a patient who resides or recently traveled to an endemic area is enough for a clinical diagnosis of LD and should be followed by treatment. The prompt administration of antibiotics during this early phase may blunt antibody production, therefore serological testing after treatment is not a reliable indicator of active infection for a subsequent treatment decision [14,15]. Similarly, some recovered individuals may have persistent antibody levels; others waning levels.

### 3.1. Indirect Testing Methods

Originally forwarded during the 1994 Conference on the Serological Diagnosis of Lyme Disease, held in Dearborn, Michigan, the two-tiered testing strategy still remains the most commonly used laboratory test for diagnostic evaluation [16]. However, this is changing since at that time it was not recognized that many antigens share common epitopes with other infections. Older, less accurate tests also utilized antigens from whole cell sonicates obtained from cultured forms of *B. burgdorferi*. It is now known that *B. burgdorferi* expresses certain antigens *in vivo* that are not found in cultured preparations. This was not realized at the time, so these *in vivo* antigens were not included in these older tests [14,15]. Additionally, the selection of antigen targets did not make use of current methodology of epitope mapping, thereby including cross-reactive targets [17]. Since then, advancements in laboratory technique have improved the clinical interpretation of these results, yet they still suffer from certain disadvantages that a single antibody test is not a measure of active infection [14,15]. Although the Centers for Disease Control and Prevention (CDC) recommends utilizing the two-tiered testing method, this is likely to change [15,18]. The traditional two-tiered method consists of two sequential tests. The first test utilizes either a sensitive Enzyme Immunoassay (EIA) or an Immunofluorescence Assay (IFA) which, if positive or indeterminate, is followed by a second, more specific serologic test, a Western blot (WB). An immunoglobulin G (IgG) WB is performed after a positive or indeterminate EIA for confirmation. An immunoglobulin M (IgM) WB is frequently performed if the patient has had symptoms for less than 30 days, commonly referred to as the “1-month rule”, but that too is changing due to problems with this method [15,18]. Human factors can also influence the interpretation of WB since it relies on visual examination, which is subjective and operator dependent. This problem has been partially, but not necessarily practically, addressed with the use of “line blots” which aid with more precise and standard densitometric blot analysis [14,15]. Yet still today, most available WB assays utilize a cultured form of *B. burgdorferi* lysates and depend on visual scoring [14,15].

On 29 July 2019, the U.S. Food and Drug Administration (FDA) approved the use of several new serologic assays that allow for a new testing strategy, a “modified two-tiered methodology”. This new strategy tries to improve upon complexities from the traditional two-tiered testing by running two sequential EIAs as the first and second tier tests and altogether avoiding the use of WBs and much interpretative variability by observers. These EIAs are designed to detect antigens expressed *in vivo* and improve specificity, but there is not much gain in sensitivity, especially during early stages of disease. One such antigen is the C6 peptide located within the variable major protein-like sequence-expressed (VlsE) locus of *B. burgdorferi* [18,19]. The OspC protein was not expressed in culture preparations and was consequently missed in older generation EIAs.

This new modified technique offers a sensitivity of 60% to 74% for the early stage of LD, 93% to 100% for the late stages of LD, and an overall specificity of 98% [16,17,18]. Despite this improvement, overall sensitivity still remains poor during the early stage of infection due to the nature of anti-borrelial antibody production [15,18,20,21,22]. The lag time of host antibody formation contributes to deficient sensitivity of these indirect tests during the first 2 weeks after tick exposure. Free circulating IgM antibodies begin to appear within 2–3 weeks and may persist for 4–6 months, while IgG antibodies may take longer to appear but can persist for years even after successful treatment. Additionally, as with all other serologic testing modalities, antibody detection from a single sample does not differentiate between active infection and past exposure. In addition, it has been found that the C6-peptide is not specific only to *B. burgdorferi* as it has cross-reactivity with other bacterial antigens [17].

A recent FDA-approved assay showed superior results compared to the EIA/WB utilized in the two-tiered system. It makes use of epitope-mapped *B. burgdorferi* antigen targets in a multiplex bead format [23]. By utilizing 10 markers, sensitivity and specificity can be tailored to marker sets that are geared towards early stage cases, and others towards later cases.

### 3.2. Direct Testing Methods

Despite improvements of serologic assays, a single test cannot diagnose a case of LD. In contrast, direct testing strategies do allow for the detection of active infection. Culture is the gold standard in other infections, but it is not practical in clinical application for LD; however, it may have research value. *B. burgdorferi* cultures utilize a highly specialized medium (Barbour–Stoenner–Kelly-H culture) that is time consuming, taking up to 8–12 weeks for interpretation and consequently not useful for a prompt clinical diagnosis [21,22].

Other direct diagnostic methods are based on the detection of antigenic material and bacterial nucleic acids. Molecular detection of *B. burgdorferi* DNA has the potential to allow for direct quantitative measurement of the actual microbe and therefore can serve as a marker of infection. These methods can be viewed as on-the-horizon techniques that may eventually be adapted for clinical use. They are especially useful for detecting genetic material that may be found circulating in the blood or cerebrospinal fluid. However, because *B. burgdorferi* replicates very slowly in comparison to other bacteria or viruses, its low abundance and few genome copies in body fluids have hampered its detection by conventional PCR testing [20,21,24]. Nonetheless, several studies have shown that *B. burgdorferi* is present in tissue and can be detected by enhancing methods, such as by utilizing isothermal amplification, multi-locus primer probes, and the use of larger starting volumes similar to those taken for blood cultures [25].

The detection of *B. burgdorferi* with the use of an antigen-capture assay has been studied but still suffers from significantly low sensitivity and specificity [21]. The quality of an antigen-capture assay depends on many variables, such as the choice of the capture and reporter of antigenic targets as well as the affinity of the antibody used for detection. Moreover, the bacterium’s antigenic inventory may vary depending on the duration of infection, which further complicates this matter.

## 4. Conclusions

In closing, direct detection methods, many of which are in development for clinical use for LD, are the most certain way to identify an active case of LD. In contrast to a validated direct test, a single antibody-based test cannot surmount the uncertainty of a positive result in an endemic area where many residents may be positive from past infections. Direct testing methods for *B. burgdorferi* may also conclusively assist in the proper evaluation of nonspecific symptoms that would otherwise lead to a misdiagnosis of LD. Future developments may change the way we approach the diagnosis of LD. However, until then, proper understanding of current serologically based methods and evolving direct testing strategies remains crucial for the most accurate interpretation that best serves our patients.

## Figures and Tables

**Table 1:** Lyme arthritis and differential diagnoses.

Differential Diagnosis	Common Pattern of Arthritis	Common Distinguishing Features
**Lyme Arthritis**	Asymmetric Monoarthrtitis or oligoarthritis Knees, shoulders, wrists, elbows, TMJ	Epidemiological exposure to *Ixodes scapularis* tick EM skin lesion in early localized stage Positive Lyme serology Synovial fluid ~25,000 WBCs/mm^3^ with neutrophilic predominance
**Oligoarticular Juvenile Idiopathic Arthritis**	Asymmetric Monoarthritis or oligoarthritis Knee, ankles, wrists	Children 2–4 years old 3x more common in girls than boys Inflammatory biomarkers usually normal or mildly elevated Positive ANA in >50% Negative RF
**Reactive Arthritis**	Asymmetric Oligoarthritis or monoarthritis Knees, ankles, feet Enthesitis and spondylitis may occur	Age 20–40 Onset within 4 weeks of urogenital or gastrointestinal infection Inflammatory biomarkers usually elevated Positive HLA-B27 in 80% of caucasian males Negative RF, Negative ANA
**Rheumatoid Arthritis**	Symmetric Polyarthritis Small joints more common than large joints Wrists, MCPs, PIPs, MTPs, ankles	Age 40–60 3x more common in females Positive RF in 80% Positive anti-CCP in 70% Inflammatory biomarkers usually elevated
**Psoriatic Arthritis**	Asymmetric Oligoarthritis or monoarthritis DIPs, PIPs, MCPs, wrists, ankles Enthesitis and spondylitis may occur	Presence of psoriasiform skin rash Nail changes (dystrophy, pitting) Negative RF, negative ANA
**Septic Arthritis**	Monoarthritis Abrupt onset of joint pain with swelling and erythema, usually associated with signs of systemic infection	Inflammatory biomarkers markedly elevated Synovial fluid usually with >50,000 WBCs/mm^3^ with neutrophilic predominance Positive synovial fluid culture in >95% Bacterial blood culture positive in 50%
**Fibromyalgia**	Widespread pain syndromeAbsence of musculoskeletal inflammation = no arthritis	Disorder of pain regulation Normal inflammatory biomarkers

ANA: anti-nuclear antibody; CCP: cyclic citrullinated peptide; DIPs: distal interphalangeal joints; EM: erythema migrans; HLA: human leukocyte antigen; MCPs: metacarpophalangeal joints; PIPs: proximal interphalangeal joints; MTPs: Metatarsophalangeal joints; RF: rheumatoid factor; TMJ: temporomandibular joints; WBCs: white blood cells.

## Data Availability

Not applicable.

## Glossary

- Arthralgia: joint pain.
- Arthritis: joint inflammation with features of pain, swelling, warmth, erythema.
- Synovitis: inflammation of the synovial membrane, a layer of connective tissue that covers a joint.
- Monoarthritis: arthritis of 1 joint at a time.
- Oligoarthritis: arthritis involving 2 to 4 joints at a time.
- Polyarthritis: arthritis of more than 4 joints at a time.
- Spondylitis: arthritis of the vertebral joints, including the sacroiliac joints.
- Enthesitis: inflammation of tendon or ligament insertion into bone, commonly of the achilles tendon. A type of periarticular manifestation.
- Tenosynovitis: inflammation of the fluid sheath that surrounds a tendon.
